# Behavioral and Psychological Effects of Coronavirus Disease-19 Quarantine in Patients With Dementia

**DOI:** 10.3389/fpsyt.2020.578015

**Published:** 2020-09-09

**Authors:** Annachiara Cagnin, Raffaele Di Lorenzo, Camillo Marra, Laura Bonanni, Chiara Cupidi, Valentina Laganà, Elisa Rubino, Alessandro Vacca, Paolo Provero, Valeria Isella, Nicola Vanacore, Federica Agosta, Ildebrando Appollonio, Paolo Caffarra, Ilaria Pettenuzzo, Renato Sambati, Davide Quaranta, Valeria Guglielmi, Giancarlo Logroscino, Massimo Filippi, Gioacchino Tedeschi, Carlo Ferrarese, Innocenzo Rainero, Amalia C. Bruni, SINdem COVID-19 Study Group, Erica Gallo

**Affiliations:** ^1^ Department of Neuroscience (DNS), University of Padua, Padua, Italy; ^2^ Regional Neurogenetic Centre, Department of Primary Care, ASP-CZ, Catanzaro, Italy; ^3^ Memory Clinic, Fondazione Policlinico Gemelli, IRCCS Università, Cattolica del Sacro Cuore, Rome, Italy; ^4^ Department of Neuroscience, Imaging and Clinical Sciences, University G. d’Annunzio of Chieti-Pescara, Chieti, Italy; ^5^ CDCD Ospedale del Delta, AUSL Ferrara, Ferrara, Italy; ^6^ Department of Neuroscience and Mental Health, AOU Città della Salute e della Scienza di Torino, Turin, Italy; ^7^ Aging Brain and Memory Clinic, Department of Neuroscience, University of Torino, Turin, Italy; ^8^ Department of Molecular Biotechnology and Health Sciences, University of Torino, Turin, Italy; ^9^ Center for Omics Sciences, IRCCS San Raffaele Scientific Institute, Milan, Italy; ^10^ Department of Medicine and Surgery and Milan Center for Neuroscience (NeuroMi), University of Milano – Bicocca, Monza, Italy; ^11^ National Institute of Health, Rome, Italy; ^12^ Division of Neuroscience, IRCCS San Raffaele Scientific Institute, Milan, Italy; ^13^ Vita-Salute San Raffaele University, Milan, Italy; ^14^ Unit of Neuroscience, University of Parma, Parma, Italy; ^15^ Department of Clinical Research in Neurology, Center for Neurodegenerative Diseases and the Aging Brain, University of Bari, Bari, Italy; ^16^ Department of Basic Medicine Neuroscience and Sense Organs, University Aldo Moro Bari, Bari, Italy; ^17^ Department of Medical and Surgical Sciences, University of Campania “Luigi Vanvitelli”, Naples, Italy

**Keywords:** behavioral and psychological symptoms, behavioral symptoms, psychological symptoms, quarantine, dementia, caregiver, coronavirus disease, gender

## Abstract

**Background:**

In March 2020, the World Health Organization declared a global pandemic due to the novel coronavirus SARS-CoV-2 and several governments planned a national quarantine in order to control the virus spread. Acute psychological effects of quarantine in frail elderly subjects with special needs, such as patients with dementia, have been poorly investigated. The aim of this study was to assess modifications of neuropsychiatric symptoms during quarantine in patients with dementia and their caregivers.

**Methods:**

This is a sub-study of a multicenter nation-wide survey. A structured telephone interview was delivered to family caregivers of patients with diagnosis of Alzheimer disease (AD), dementia with Lewy bodies (DLB), frontotemporal dementia (FTD), and vascular dementia (VD), followed regularly at 87 Italian memory clinics. Variations in behavioral and psychological symptoms (BPSD) were collected after 1 month since quarantine declaration and associations with disease type, severity, gender, and caregiver’s stress burden were analyzed.

**Results:**

A total of 4,913 caregivers participated in the survey. Increased BPSD was reported in 59.6% of patients as worsening of preexisting symptoms (51.9%) or as new onset (26%), and requested drug modifications in 27.6% of these cases. Irritability, apathy, agitation, and anxiety were the most frequently reported worsening symptoms and sleep disorder and irritability the most frequent new symptoms. Profile of BPSD varied according to dementia type, disease severity, and patients’ gender. Anxiety and depression were associated with a diagnosis of AD (OR 1.35, CI: 1.12–1.62), mild to moderate disease severity and female gender. DLB was significantly associated with a higher risk of worsening hallucinations (OR 5.29, CI 3.66–7.64) and sleep disorder (OR 1.69, CI 1.25–2.29), FTD with wandering (OR 1.62, CI 1.12–2.35), and change of appetite (OR 1.52, CI 1.03–2.25). Stress-related symptoms were experienced by two-thirds of caregivers and were associated with increased patients’ neuropsychiatric burden (p<0.0001).

**Conclusion:**

Quarantine induces a rapid increase of BPSD in approximately 60% of patients and stress-related symptoms in two-thirds of caregivers. Health services need to plan a post-pandemic strategy in order to address these emerging needs.

## Introduction

An outbreak of a novel coronavirus (severe acute respiratory syndrome coronavirus 2 -SARS-CoV-2) emerged in Wuhan, China, in late 2019 and spread to Europe in February 2020 with the first infected patient diagnosed in Italy. It has since then spread globally, with over 10 million confirmed cases as of June 30, 2020. SARS-CoV-2 has been identified as the cause of COVID-19, a respiratory illness with heterogeneous systemic and neurological symptoms (1–3). Older adults and subjects with higher comorbidities have the lower prognosis (4) and presence of dementia increases the risk of mortality after COVID-19 (5). For the containment and management of COVID-19, government authorities have introduced mitigation strategies based on measures of lockdowns, travel restrictions, and mass quarantine. Italy was the first European Country to impose a nationwide lockdown on March 13, 2020.

Confinement and isolation have been proven to be highly effective for the control of infectious diseases, including COVID-19 pandemic (6). However, previous outbreaks of SARS and MERS showed that quarantine has a negative effect on mental health, with increased psychiatric symptoms particularly related to stress reactions such as anxiety, depression, and anguish (7). Considering findings from previous outbreaks and preliminary observations during the COVID-19 pandemic, the scientific community has launched an alarm about a possible imminent “pandemic” of psychiatric disorders (8–10). Factors triggering an increase of post-pandemic psychiatric disorders may be multiples. Importance has been given to a direct effect of isolation, with restrictions on movements, impoverishment of social contacts, and affective relationships, perceived loneliness. Anxiety may arise from the rapid need to adapt to new lifestyle and changes of day to day routines. In addition, an increased state of alert due to fear of contagion and grief or even mourning for the loss of family members or friends for COVID-19 may undermine mental health wellbeing (10).

These considerations apply to the general population and very few information is available for the most vulnerable persons in society, such as elderlies and those affected by dementia (11, 12). Individuals with dementia are frail, dependent on caregivers for daily living activities and needing the support of a network of social and health services resources (memory clinics, Alzheimer café, diurnal centers, physiotherapy, etc.). In this scenario, extended lockdown with imposed self-isolation and change or deprivation of usual daily activities may represent a stressor event in both patients and caregivers with high risk to induce anxiety and depression (13). Changes in neuropsychiatric symptoms in subjects with dementia may exacerbate the psychological effects of lockdown in their caregivers, situation which may further worsen patients’ behavioral symptoms, acting in a vicious loop of mutual increase of psychiatric burden. Finally, confinement reduces access to physical exercise or even physiotherapy, and movement restriction exacerbates symptoms of dementia (13, 14). In turn, lack of activities and global cognitive and physical stimulation may cause delirium in individuals with dementia, contributing further to morbidity. There is also increase evidence that psychological symptoms due to stressor events can contribute to cognitive decline (15).

A call of action for a plan to evaluate and counteract mental status illnesses in the COVID-19 post-pandemic phase in the general population has been launched (16). However, knowledge on the psychological effects of quarantine in patients with dementia, at higher risk of mental health worsening, is lacking. In this perspective, the aim of this study was to investigate the frequency and type of changes in behavioral and psychological symptoms of dementia (BPSD) during the first month of COVID-19 quarantine in patients with different types of brain diseases leading to dementia and the psychological effects in their caregivers. Factors that may modulate the change in neuropsychiatric symptoms such as disease type and severity, patient’s gender, and caregiver’s stress were also investigated.

## Methods

This is an observational sub-study nested in a larger multicenter nation-wide survey conducted in Italy between 14 and 24 April 2020 and evaluating the effects of quarantine due to COVID-19 pandemic on cognitive, behavioral, and motor symptoms of patients with dementia, impact of quarantine in family caregivers, and changes of health services devoted to dementia care. Here we report results regarding patients’ BPSD changes and caregivers’ psychological symptoms.

### Study Protocol

Eighty-seven Centers for Cognitive Disorders and Dementia (CDCD) equally distributed among Northern, Center, and Southern Italy were recruited. Invitation to participate in the survey was made through two Italian scientific societies involved in dementia care and research, the Italian Neurological Society for Dementia (SINdem), and the Italian Association of Psychogeriatrics (AIP), to all their participants working in the CDCD. Eighty-nine centers responded positively and two centers were not able to conclude the enrolment and therefore 87 finally participated in the study. Patient’s response rate was 98%. Study collaborators of each CDCD were asked to deliver by phone call a semi-structured interview to family caregivers of patients with dementia on regular clinical follow-up. Inclusion criteria were a) a diagnosis of one of the most common forms of dementia including: 1. Alzheimer’s disease (AD), 2. dementia with Lewy bodies (DLB), 3. frontotemporal dementia (FTD), and 4. vascular dementia (VD); b) presence of a family caregiver. Exclusion criteria included a diagnosis of mild cognitive impairment and primary psychiatric disorders. The semi-structured interview was administered to family caregivers through a questionnaire divided in two parts, regarding patients’ and caregivers’ features (**Supplementary Material**). The part related to patients consisted of nine questions regarding modifications of dementia-related symptoms after beginning of quarantine and clinical data on previous physical independence and awareness of current pandemic. In particular, caregivers were asked whether patients had worsened one or more of the following BPSD: irritability, apathy, agitation, anxiety, depression, sleep disturbances, aggressiveness, wandering, appetite change, hallucinations, and delusions. In addition, the onset of new symptoms among the abovementioned BPSD was enquired. A further question about the need of drug treatments modifications due to worsened or new BPSD was administered. The part related to family caregivers explored 16 domains concerning demographic and social characteristics, life style and work changes after quarantine, use of medical care and health services for patients needs, and psychological effects of pandemic. Each center was asked to practice with the telephone-based interview before starting recruiting. A person of the organizing committee was available to solve questions or doubts risen from initial training. No formal harmonized procedure of delivering the interview was planned.

The study was initially approved by the Ethics Committee of the Coordinating Center (University of Torino on April 7, 2020, n.00150/2020) and by the local ethics boards. Participants gave informed consent to the study.

### Statistical Analysis

The primary data source consists of all the interviews administered (total sample = 4,913). A sub-sample of patients with BPSD changes (n= 2,929) was extracted, considering patients with BPSD changes having either preexisting and/or new-onset symptoms. The fields with missing values are approximately 0.6% for which no substitution has been made.

EPI Info 7.2 software (EPIINFO ™, CDC, Atlanta, USA) was used for the statistical analysis. Microsoft Excel (Microsoft Office 2019) was used to process the charts. Microsoft Access (Microsoft Office 2019) was used to create the intermediate analysis tables. The analysis of the descriptive frequencies and the crude univariate logistic regression for the preexisting and new BPSD symptoms were performed, stratifying where necessary to control the confounding’s. Subsequently, unconditional and matched logistic regressions were performed to assess the dependence on the diagnosis, the degree of disease severity and gender, setting the confidence limits at 95%.

## Results

### Patients’ Demographic and Clinical Characteristics

Data were obtained through interview of 4,913 caregivers of patients with dementia after a mean quarantine period of 47.2 ± 6.4 days. Patients’ demographic, social, and clinical characteristics are reported in **Table 1**.

**Table 1:** Demographic and clinical characteristics of patients affected by dementia.

Patients	Total (n = 4913)	AD (n = 3372)	DLB (n = 360)	FTD (n = 415)	VD (n = 766)
Age (years, mean ± SD)	78.3 ± 8.2	78.3 ± 8	78 ± 7.3	72.3 ± 8.9	81.6 ± 7
Gender, female % (n)	59.7 (2.934)	63.5 (2.140)	42.2 (153)	46.7 (194)	58.4 (447)
Disease duration (years, mean ± SD)	4.5 ± 3.1	4.6 ± 3.1	4.5 ± 3	4.8 ± 3.2	4.1 ± 2.9
Regional distribution in Italy % (n)					
North	32.2 (1582)	26.5 (892)	35.3 (127)	47.5 (197)	47.8 (366)
Center	31.5 (1550)	34.1 (1151)	36.4 (132)	21.2 (88)	23.4 (179)
South/Islands	36.3 (1781)	39.4 (1328)	28.3 (102)	31.3 (130)	28.8 (221)
CDR stage % (n)					
1	25.0 (1222)	24.3 (816)	26.3 (94)	23.4 (96)	28.4 (216)
2	47.8 (2334)	49.2 (1651)	41.9 (151)	48.6 (199)	43.8 (333)
3	27.2 (1325)	26.5 (885)	31.8 (114)	28.0 (115)	27.8 (211)
Worsening of BPSD, yes % (n)	51.9 (2542)	50.5 (1699)	63.8 (229)	55.3 (229)	50.3 (385)
Gender, female % (n)	57.9 (1472)	62.9 (1068)	38.4 (88)	45.4 (104)	55.1 (212)
Onset of new BPSD, yes % (n)	25.9 (1272)	26.7 (901)	23.3 (84)	21.9 (91)	25.6 (196)
Gender, female % (n)	56.7 (722)	59.8 (539)	41.7 (35)	41.8 (38)	56.1 (110)
BPSD-related drug modification, yes % (n)	27,6 (795)	25,9 (505)	33,6 (83)	32,1 (78)	29,1 (129)

AD, Alzheimer’s disease; DLB, dementia with Lewy bodies; FTD, frontotemporal dementia; VD, vascular dementia; CDR, clinical dementia rating; BPSD, behavioral and psychological symptoms of dementia.

Patients had a diagnosis of AD in 69% of cases, VD in 16%, FTD in 8%, and DLB in 7%. Mean age, disease duration, disease severity, gender type, and geographical distributions of recruiting centers were not different between disease groups.

### Neuropsychiatric Symptoms Changes

Caregivers reported BPSD changes (worsening and/or new onset BPSD) in 2,929 patients (59.6%) after 1-month from beginning of quarantine. Worsening of preexisting BPSD was described in 51.9% of cases. The DLB group had the highest frequency of increased BPSD (63.8%), followed by FTD (55.3%), AD (50.5%), and VD (50.3%). Onset of new BPSD was reported in 25.9%, with higher frequency in AD (26.7%) and lower in FTD (21.9%) (**Table 1**).

**Figure 1:**
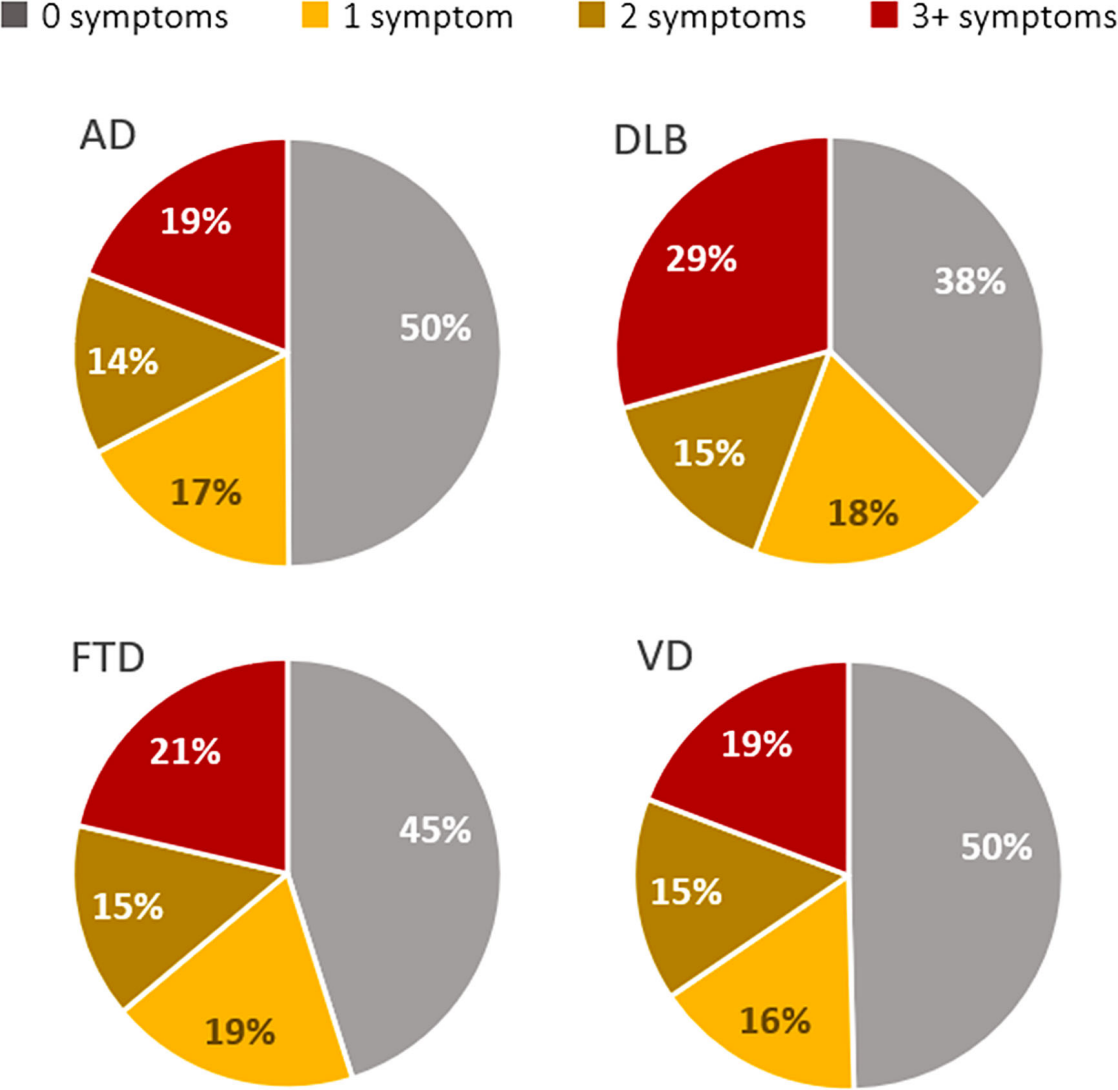
Distribution of classes of behavioral and psychological symptoms (BPSD) burden defined as number of neuropsychiatric symptoms during quarantine divided by disease type.

Patients with DLB and BPSD changes had a wider burden of neuropsychiatric symptoms (considering both worsened and new symptoms) with almost 30% having three or more symptoms, respect to FTD (21%) and AD and VD (both 19%) (**Figure 1**).

The increased burden of BPSD required a modification of drug treatments in 27.6% of patients with BPSD changes. In the multivariate analysis frequency of neuropsychiatric symptoms was not associated with patient’s age, gender, type of dementia, severity, and duration of the disease.

**Figure 2:**
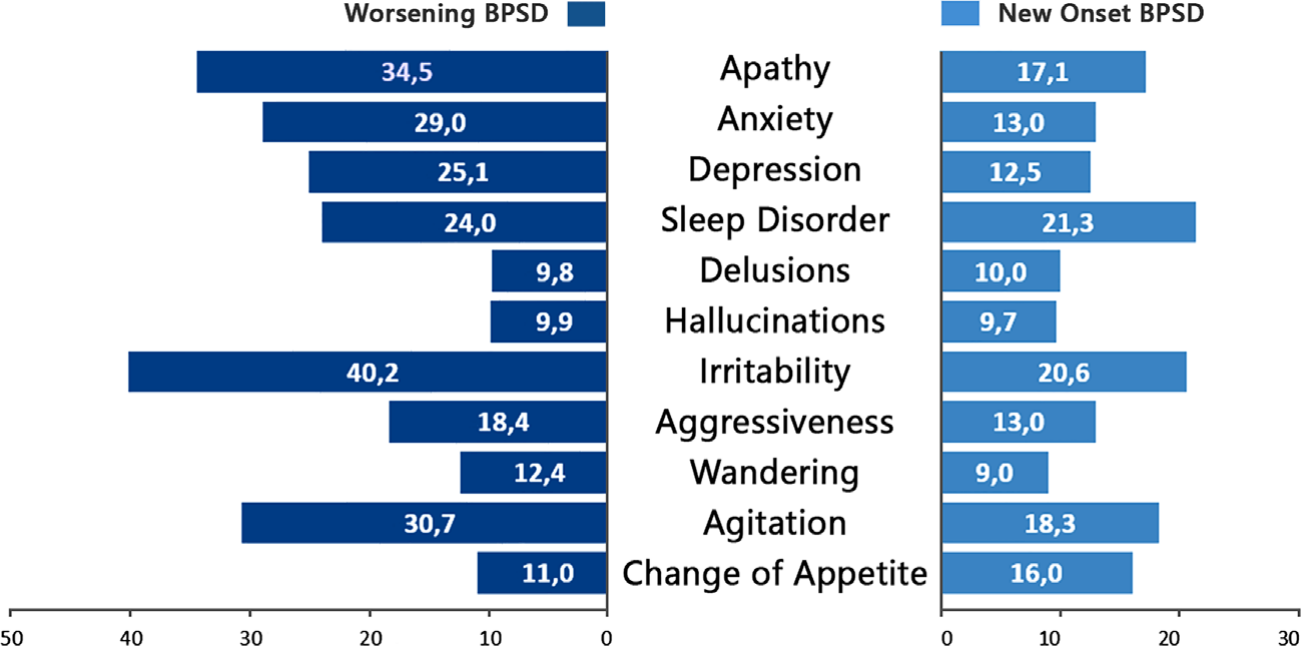
Frequency of behavioral and psychological symptoms (BPSD) worsened (dark blue) and newly ongoing (light blue) in the sample of patients with BPSD changes (worsened and/or new onset, n = 2,929).

As far as the type of BPSD, the most frequently reported worsened BPSD was irritability (40.2%), followed by apathy (34.5%), agitation (30.7%), anxiety (29%), depression (25.1%), and sleep disorder (24%). The less frequently reported BPSD were in the psychotic domain, with both hallucinations and delusions worsened in 10% of cases. Sleep disorder and irritability were the main newly onset BPSD during pandemic (**Figure 2**). In **Table 2** are reported the prevalence of worsened and new BPSD in the entire patients’ sample.

**Table 2:** Frequency distribution of worsened preexisting and new behavioral and psychological symptoms of dementia (BPSD) in the entire patients’ sample and divided by disease type.

Patients	Total (n = 4,913)	AD (n = 3,372)	DLB (n = 360)	FTD (n = 415)	VD (n = 766)
Worsening, % (n):					
Apathy	17.9 (881)	17.8 (601)	21.9 (79)	19.8 (82)	15.5 (119)
Anxiety	15.1 (744)	15.7 (529)	18.6 (67)	12.5 (52)	12.5 (96)
Depression	13.0 (640)	12.4 (418)	18.0 (65)	12.0 (50)	14.0 (107)
Sleep disorder	12.5 (615)	11.5 (388)	21.9 (79)	13.5 (56)	12.0 (92)
Delusions	5.1 (251)	4.4 (149)	10.0 (36)	6.3 (26)	5.2 (40)
Hallucinations	5.1 (252)	4.1 (139)	18.6 (67)	3.4 (14)	4.2 (32)
Irritability	20.9 (1026)	20.6 (694)	20.2 (73)	21.7 (90)	22.1 (169)
Aggressiveness	9.6 (470)	8.9 (301)	10.2 (37)	11.1 (46)	11.2 (86)
Wandering	6.4 (315)	6.1 (204)	3.9 (14)	10.1 (42)	7.2 (55)
Agitation	16.0 (784)	15.0 (505)	20.5 (74)	19.5 (81)	16.2 (124)
Change of appetite	5.7 (282)	5.3 (178)	6.1 (22)	8.7 (36)	6.0 (46)
New onset, % (n)					
Apathy	4.4 (218)	4.6 (154)	4.4 (16)	3.9 (16)	4.2 (32)
Anxiety	3.4 (165)	3.5 117)	3.3 12)	2.4 10)	3.4 26)
Depression	3.2 (159)	3.5 (119)	1.7 (6)	1.7 (7)	3.5 (27)
Sleep disorder	5.5 (271)	5.7 (191)	3.0 (11)	4.6 (19)	6.5 (50)
Delusions	2.6 (127)	2.4 (81)	3.3 (12	2.4 (10)	3.1 (24)
Hallucinations	2.5 (124)	2.6 (88)	2.8 (10)	1.4 (6)	2.6 (20)
Irritability	5.4 (263)	5.8 (194)	4.2 (15)	3.6 (15)	5.1 (39)
Aggressiveness	3.4 (166)	3.4 (113)	3.0 (11)	3.4 (14)	3.7 (28)
Wandering	2.3 (115)	2.4 (82)	1.1 (4)	1.9 (8)	2.7 (21)
Agitation	4.7 (233)	5.0 (169)	3.9 (14)	3.6 (15)	4.6 (35)
Change of appetite	4.1 (203)	4.4 (148)	2.8 (10)	3.9 (16)	3.8 (29)

AD. Alzheimer’s disease; DLB, dementia with Lewy bodies; FTD, frontotemporal dementia; VD, vascular dementia; BPSD, behavioral and psychological symptoms of dementia.

**Figure 3:**
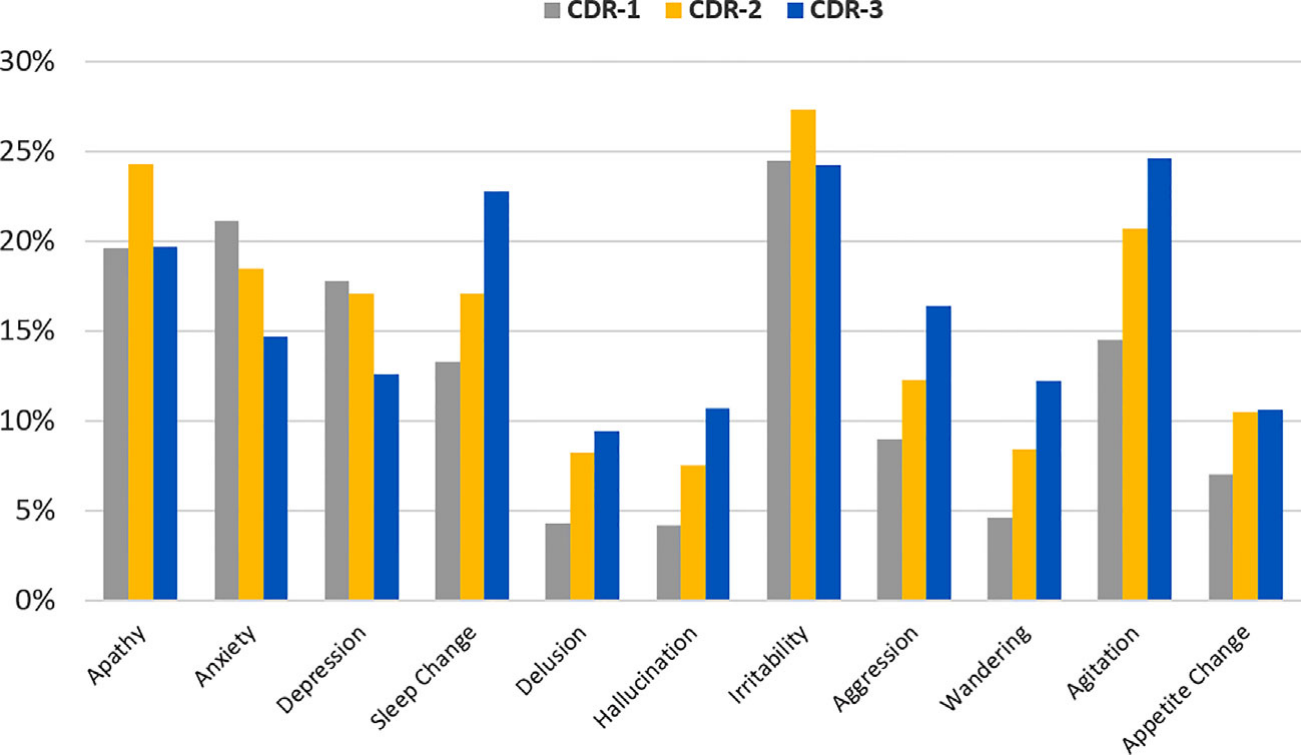
Frequency of neuropsychiatric symptoms in patients with behavioral and psychological symptoms (BPSD) changes (worsened and/or new onset, n=2,929) divided by disease severity defined by Clinical Dementia Rating scale (CDR): mild: CDR-1 gray bar; moderate: CDR-2 orange bar and severe: CDR-3 blue bar.

Prevalence of increased BPSD changes (worsened and/or new symptoms) was similar across different classes of disease severity defined by the Clinical Dementia Rating scale (CDR): mild= CDR-1: 55.8%; moderate= CDR-2: 62.3%; severe CDR-3: 58.6%). These results were maintained analyzing separately preexisting and new BPSD. Instead, the type of BPSD changes varied according to disease severity. Frequency distributions of specific BPSD by CDR severity is represented in **Figure 3**. Anxiety was most frequent in patients with mild dementia while agitation and sleep disorder in patients with severe dementia.

**Figure 4:**
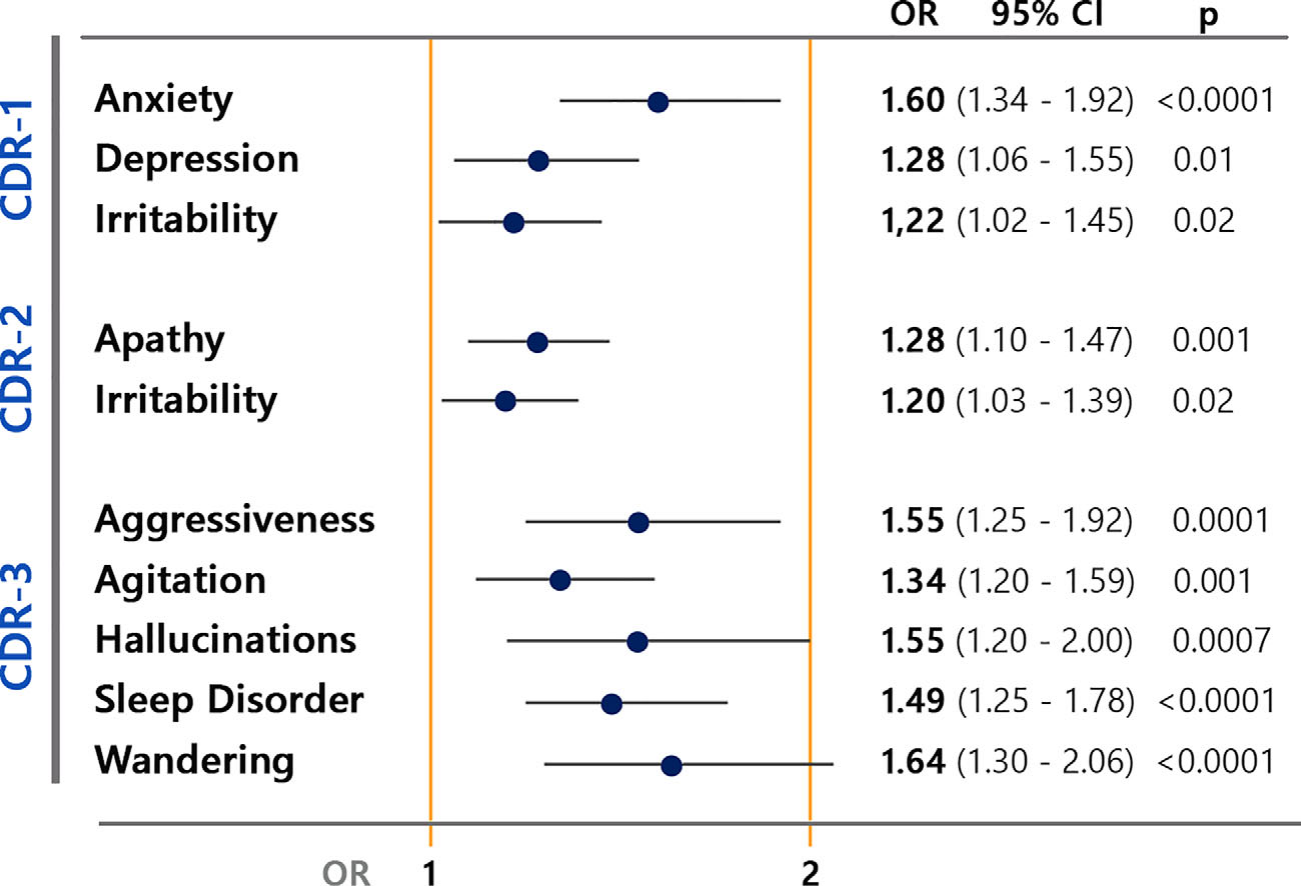
Multivariate analyses of behavioral and psychological symptoms (BPSD) changes associated to disease severity defined by CDR (Clinical Dementia Rating) in mild (CDR-1), moderate (CDR-2), and severe (CDR-3).

Results of the multivariate analyses of neuropsychiatric symptoms in different classes of disease severity showed an increased risk of a wider pattern of BPSD in patients with severe disease, while anxiety was associated with mild disease severity (**Figure 4**).

### Profile of Neuropsychiatric Symptoms Changes and Disease Type

**Figure 5:**
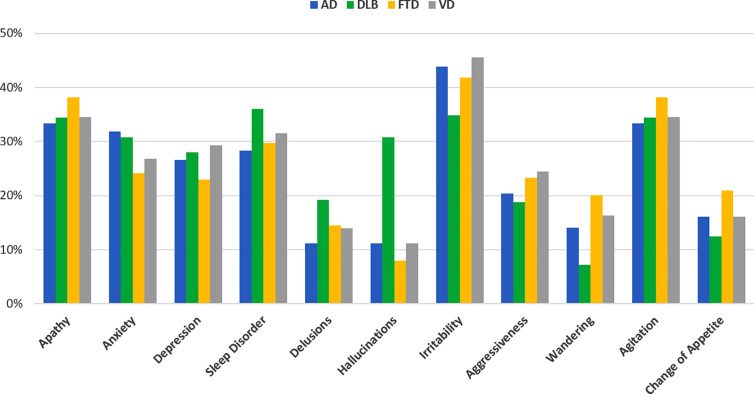
Frequency of neuropsychiatric symptoms in patients with behavioral and psychological symptoms (BPSD) changes (worsened and/or new onset, n=2,929) divided by disease type (blue bar AD, green DLB, yellow FTD, gray VD).

Type of dementia was associated with different frequency distribution of specific neuropsychiatric symptoms (**Figure 5**).

Worsening of sleep disorder and hallucinations were more frequent in DLB compared to other types of dementia, while worsening of wandering and change of appetite were more frequently reported in FTD (**Table 2** and **Supplementary Table 1**). Anxiety was more frequently reported in AD and DLB than in FTD and VD. On the contrary, some symptoms increased similarly across disease groups such as apathy.

**Figure 6:**
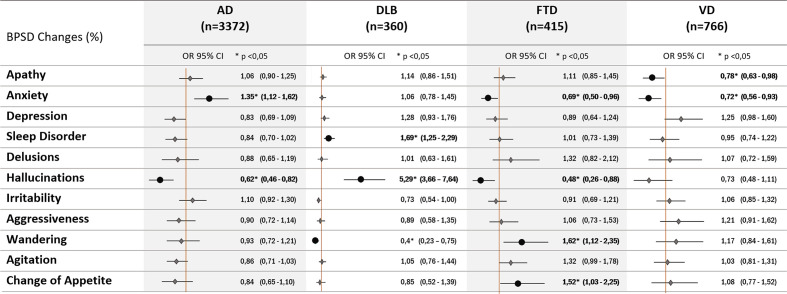
Multivariate analyses of worsened neuropsychiatric symptoms associated to disease types (diagnosis of AD, Alzheimer disease; DLB, dementia with Lewy bodies; FTD, frontotemporal dementia; VD, vascular dementia).

In the multivariate analyses the risk profiles of increased BPSD were different according to type of dementia (**Figure 6**). Having AD was associated with an increased risk of anxiety, DLB with hallucinations and sleep disorder, and FTD with wandering and change of appetite. On the opposite, AD and FTD had lower risk of worsening hallucinations and FTD and VD to develop worsening of anxiety. No significant associations were found between type of dementia and type of new BPSD.

### Profile of Neuropsychiatric Symptoms Changes and Gender

**Figure 7:**
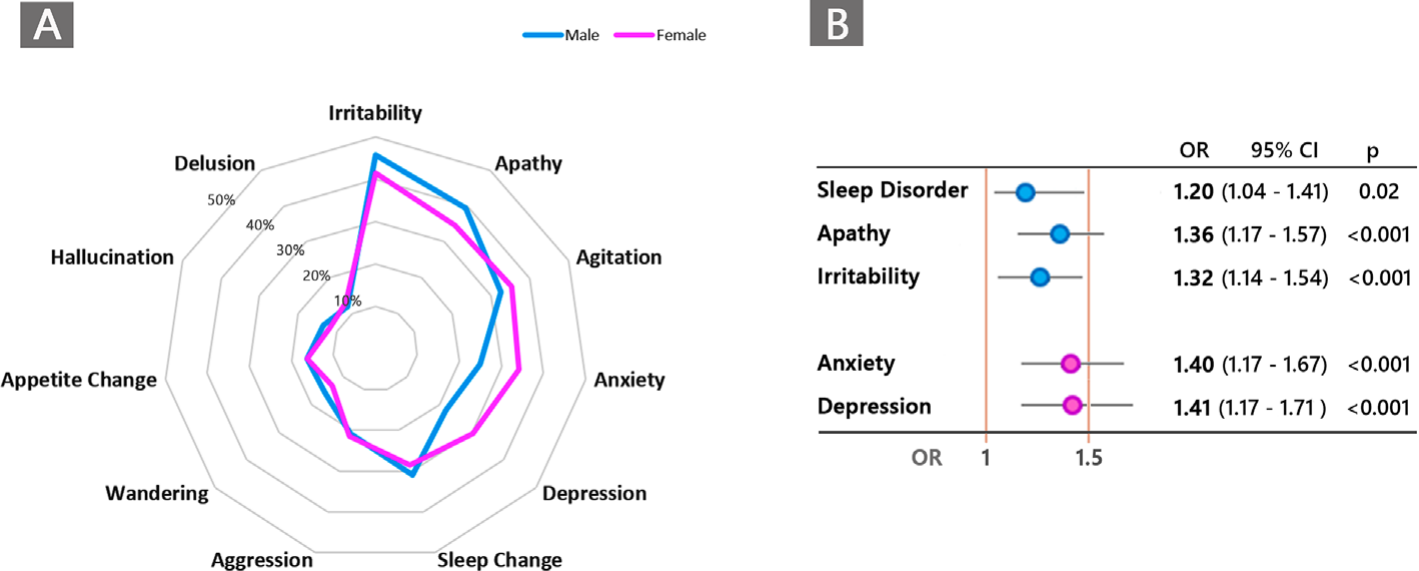
**(A)** Graphical representation of frequency distribution of neuropsychiatric symptoms according to male (blue) and female (violet) gender in patients with behavioral and psychological symptoms (BPSD) changes (worsened and/or new onset, n=2,929). **(B)** Type of neuropsychiatric symptoms significantly associated with male gender (blue color) and female gender (violet) in the entire population of patients with dementia.

Gender influenced the type of BPSD worsened during quarantine. Symptoms of anxiety and depression were more frequently reported in female patients, while apathy and irritability in male patients (p<0.05) as shown in **Figure 7**.

In the multivariate analyses, increased risk of anxiety and depression was significantly associated with female gender, while apathy, irritability, and sleep disorder with male gender (**Figure 7**).

**Figure 8:**
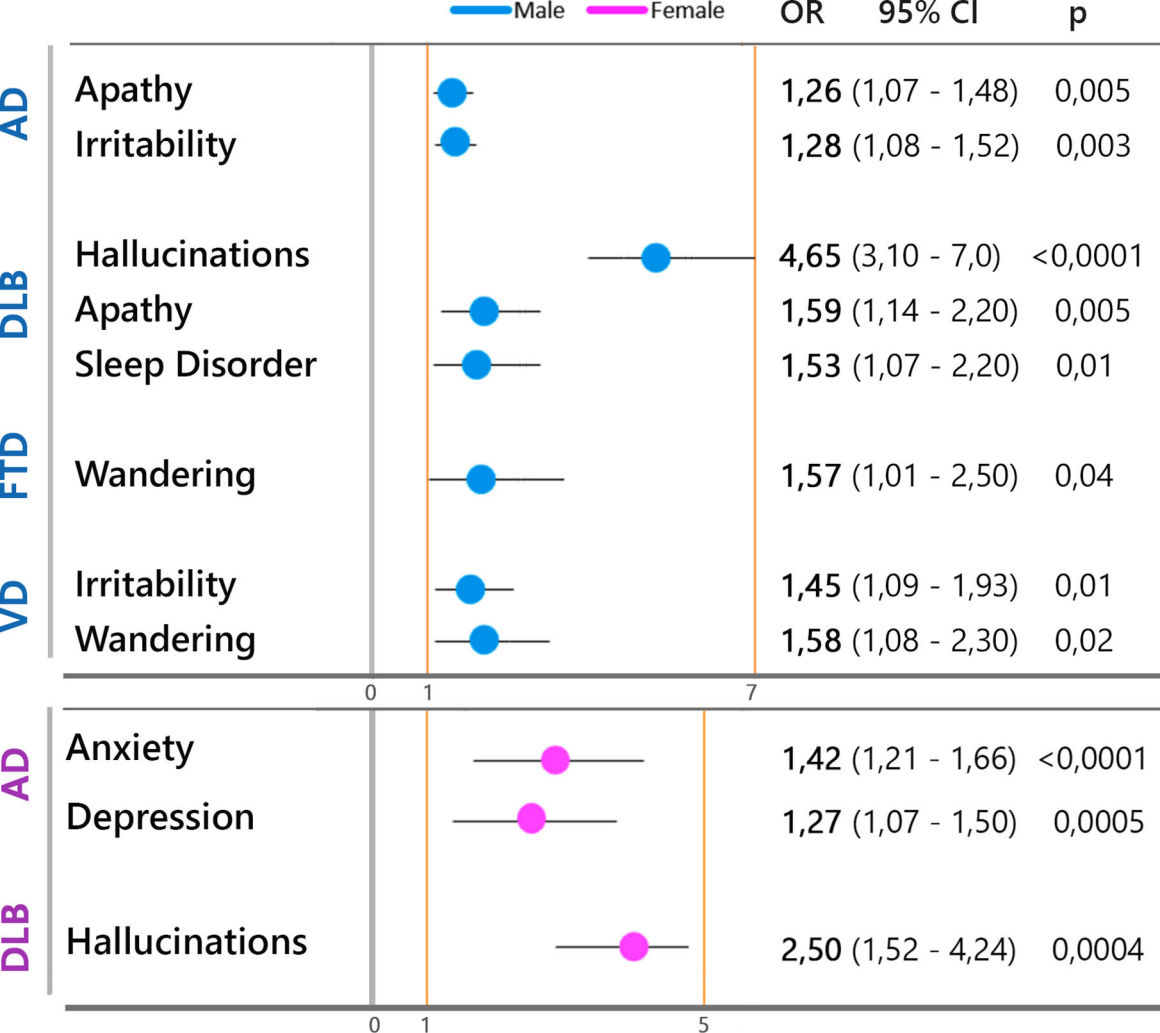
Type of neuropsychiatric symptoms significantly associated with male gender (blue circles) and female gender (violet circles) by disease type.

Within the disease group, different frequency of specific neuropsychiatric symptoms was observed in females compared to males. Gender risk of BPSD by disease types showed different associations which are summarized in **Figure 8**. In AD, the risk of increased anxiety and depression was associated with being female patients, while the risk of apathy was associated with male patients. In DLB a higher risk of increased hallucinations was associated with male gender, and sleep disorder.

### Caregivers’ Psychological Changes

Demographic, social, and psychological data of caregivers are summarized in **Table 3**.

**Table 3:** Demographic, social, and psychological characteristics of family caregivers.

Caregivers	Total (n = 4913)	AD (n = 3372)	DLB (n = 360)	FTD (n = 415)	VD (n = 766)
Age (years, mean ± SD)	59.3 ± 13	59.3 ± 13.1	60.7 ± 12.7	59.1 ± 13.6	60 ± 12.4
Gender, female % (n)	53.9 (2649)	51.2 (1724)	66.4 (240)	55.4 (230)	59.4 (455)
Cohabitant caregiver, % (n)	58.9 (2876)	58.1 (1945)	63.5 (228)	69.6 (288)	54.4 (415)
Presence of housemates, % (n)	63.3 (3076)	63.1 (2104)	62.8 (224)	58.8 (241)	67.0 (507)
Degree of kinship, % (n)					
Spouses	36.0 (1739)	35.0 (1160)	43.1 (154)	54.8 (221)	26.9 (204)
Son/daughter	54.5 (2636)	55.5 (1840)	48.7 (140)	37 (149)	62.5 (473)
Others	9.5 (460)	9.5 (318)	8.2 (29)	8.2 (33)	10.6 (80)
Change of conflicts, % (n)					
Increased	22.6 (1105)	21.9 (735)	23.4 (84)	28.7 (119)	21.8 (167)
Decreased	8.0 (394)	8.5 (285)	7.2 (26)	7.0 (29)	7.1 (54)
Concern of patient’s health, % (n)	75.1 (3662)	75.2 (2518)	76.6 (272)	74.0 (304)	74.6 (568)
Stress-related feelings (%)					
Anxiety	45.9 (2242)	46.1 (1543)	43.4 (155)	44.2 (182)	47.4 (362)
Depression	18.6 (902)	17.2 (573)	21.3 (76)	24.3 (101)	20.3 (152)
Anguish	29.3 (1422)	28.4 (943)	28.9 (103)	32.1 (133)	32.2 (243)
Irritability	26.4 (1285)	25.3 (843)	30.4 (109)	28.3 (117)	28.5 (216)
Abandonment	22.0 (1072)	21.2 (711)	22.2 (80)	24.8 (102)	23.6 (179)
Helplessness	34.2 (1672)	34.3 (1150)	33.0 (118)	33.7 (139)	34.9 (265)

AD, Alzheimer’s disease; DLB, dementia with Lewy bodies; FTD, frontotemporal dementia; VD, vascular dementia.

During quarantine a large range of stress-related feelings were reported by 65.9% (n=3,240) of caregivers. Almost 46% had symptoms of anxiety, followed by helplessness (34.2%), anguish (29.3%), irritability (26.4%), abandonment (22%), and depression (18.6%). There were not differences in frequency distribution of caregiver’s psychological symptoms across types of dementia.

Being females conferred an increased risk to develop anxiety (OR 1.4, CI 1.3–1.6, p<0.0001), anguish (OR 1.5, CI 1.2–1.7, p<0.0001), helplessness (OR 1.2, CI 1.1–1.4, p<0.01). Among social characteristics, living with housemates reduced the caregiver’s risk to develop symptoms of depression (OR 1.6, CI 0.5–0.7) and to conflict with the patient (OR 0.7, CI 0.6–0.8) (p<0.0001).

Presence of at least one caregiver’s stress-related symptom was associated with increased risk of worsened preexisting BPSD (OR 2.6, CI 2.3–13) and onset of new BPSD (OR 1.6, CI 1.4–1.9) (p<0.0001).

## Discussion

In this nation-wide survey performed in Italy after 1 month from the beginning of COVID-19 quarantine an increased burden of neuropsychiatric symptoms was reported in approximately 60% of community-dwelling persons affected by dementia by their family caregivers. Treatment drug modifications were made in 27.6% of these patients. The most frequently reported BPSD were symptoms of the anxiety-affective cluster. Profiles of BPSD changes were influenced by type of dementia, disease severity, and gender. Anxiety and depression were associated with a diagnosis of AD, mild disease severity, and female gender. Having DLB increased the risk of worsening hallucinations and sleep disorder, while FTD increased the risk of aberrant motor behavior and change of appetite. Increased BPSD burden was also associated with manifestation of psychological symptoms of distress in two-thirds of caregivers. To our knowledge this is the first survey assessing the impact of pandemic quarantine on the mental health status of a large population of patients with dementia and their caregivers.

### Pandemic Quarantine as Stressor Event

Studies on mental health modifications induced by COVID-19 pandemic in healthy subjects demonstrated increased symptoms of anxiety and depression (17–19). By now very few data are available for persons with special needs and increased fragility as patients with dementia (20). A recent study evaluated BPSD changes after 5 weeks of COVID-19 quarantine through the Neuropsychiatric Inventory questionnaire in 40 patients with AD: 20 with MCI and 20 with mild dementia (21). Worsening of BPSD respect to baseline pre-lockdown assessment was reported in 30% of patients and significant changes were found for apathy (in both groups), anxiety in MCI, agitation, and aberrant motor behavior in mild AD. We found a higher prevalence of increased BPSD respect to what has previously been reported. In our study, family caregivers were enquired about any perceived changes of patients’ neuropsychiatric symptoms; we did not use a quantitative BPSD assessment and did not compare results with a previous level of BPSD burden. Furthermore, diseases with high risk of behavioral disorders such as FTD and DLB have been included. Therefore, the higher burden of BPSD in our study may be due to different study methodology and inclusion of types of dementia other than AD. On the other hand, our results confirmed the preliminary findings that apathy, agitation, and anxiety are among the most frequently reported worsening symptoms during quarantine in patients with dementia.

In our study we found increased neuropsychiatric symptoms that rely on two different dimensions: those that represent a behavioral reaction to quarantine and those that represent an increased level of those symptoms that are specific in the different types of dementia. Increased symptoms of the anxiety-affective cluster were common (prevalence ≥ 30% for worsened irritability, agitation, and apathy and ≥ 20% of new onset sleep disorder and irritability) and were homogeneously reported across disease types. This finding is in line with many observations of increased psychological symptoms of anxiety and depression during COVID-19 quarantine in healthy subjects and give support to the notion that quarantine acts as a stressor event that induces symptoms similar of those reported in the post-traumatic stress disorder (PTSD) (22–24). Indeed, quarantine due to pandemic involves different social, emotional, psychological, and physical modifications, each one with a potential contribution to increase distress. Quarantine determines social isolation and feeling of loneliness, conditions which have been demonstrated to induce psychiatric and physical alterations in healthy individuals (25, 26). The pandemic in itself can contribute to trigger fear and contagion phobia. In persons with dementia, the increase of anxiety-related BPSD during quarantine may be interpreted as a response to a stressor event and represent a PTSD-like condition. A confirm of such speculation derives from the observation that anxiety and depression increased more in patients with mild to moderate level of severity that could still present a post-traumatic reaction. Patients with dementia have pre-trauma risk factors for the development of PTSD such as increased central nervous system sensitization due to preexisting anxiety and hyperarousal, and lower hippocampal volume (27). Neuroimaging studies showed that the neuro-anatomical correlates of PTSD are decreased volume of the hippocampus and anterior cingulate cortex which are target regions of neuropathology in AD, DLB, and FTD (27). On the other hand, there are emerging evidence of a higher risk to develop cognitive decline in patients with PTSD (15).We could here hypothesize that patients with mild to moderate level of dementia are at higher risk respect to healthy subjects to manifest a variety of anxiety-related symptoms triggered in response to isolations and restrains imposed by quarantine and that this condition may render these patients more vulnerable to the development of a PTSD-like symptomatology. This in turn might potentially worsen the trajectory of cognitive decline.

### Pandemic Quarantine as a Model of “Deprivation Syndrome”

In the last years the research field of dementia has invested a lot in demonstrating the value of cognitive, social, and physical stimulation in the prevention of cognitive decline, the modulation of the trajectory of clinical worsening in the early stage of the disease and containment of the neuropsychiatric burden (28–30). Based on the results of these studies many countries have started population programs of multimodal stimulation for persons at risk of dementia or with initial cognitive decline (31). During quarantine any formal and informal cognitive stimulation programs have been suddenly stopped. In addition, informal multidimensional stimulation derived by performances of outdoor day to day routines and maintenance of social contacts have been also markedly reduced for everyone.

Reduction of social contacts, cognitive stimulation, and physical activity can be viewed as a paradigm of “de-stimulation” or even as a model of “deprivation syndrome.” The effects of environmental deprivation defined as lack of inputs from the environment have been studied in young and adolescents for which deprivation influences subsequent psychopathology and alters cognitive developmental abilities (32, 33). Translating this term into old-age psychiatry and applying it to the topic of our study, quarantine may be viewed as an ecological experiment on the effects of acute interruption and deprivation of social, cognitive, and physical stimulation. This condition may affect cognitive and physical domains but also neuropsychiatric symptoms, reverting the well-known effects on global health of multidimensional stimulation. Obviously, this condition might be considered a sort of “partial deprivation” as family members continue to play an important, although limited, role on the social interaction with demented subjects. We can hypothesize that increase apathy, observed in approximately 35% of patents with BPSD changes and equally distributed across the disease types, might be a surrogate manifestation of a complex and global interaction of cognitive, physical, and emotional down-regulation. Apathy, in fact, may have a cognitive, emotional, and physical component and each type of apathy has defined neuro-anatomical correlates targeting prefrontal, dorsolateral, and motor cortex other than striatum (34, 35).

### Modulators of the Profile of Behavioral and Psychological Symptoms of Dementia Changes

Factors modulating the profile of increased BPSD were disease type, disease severity, and gender. Although worsening of some BPSD such as irritability and apathy are transdiagnostic, the type of neurodegenerative disease confers different risk of specific BPSD changes, such as hallucinations and sleep disorder for DLB or appetite change for FTD. Exposure to stressful events can therefore increase those neuropsychiatric symptoms for which patients with dementia are inherently more vulnerable due to the neuropathology of dementia.

Presence of visual hallucinations and alterations of sleep and wake are specific features of DLB (36). In DLB there is a higher burden of behavioral symptoms than in AD and high frequency of anxiety and depression symptoms (37, 38). On a substrate of a disease targeting the sleep-wake cycle and attentional abilities, the increase of stress-related symptoms may further worsen an efficient sleep pattern and impair attention and reality monitoring checking, with subsequent increase of hallucinations.

As regard as FTD, eating disorders are key symptoms in the diagnosis of the behavioral variant FTD, are disease specific, and are characterized by changes in dietary preferences toward carbohydrates, increased appetite, binge-eating behavior, and altered eating habits (39). We recognize the limit of this study related to the genericity of the question investigating changes of appetite without specifying whether it was a variation of increase or decrease appetite. Aberrant motor behavior may be explained as expression of reduced inhibitory control, lack of adherence to imposed societal rules and poor judgment of risks.

Disease severity was not associated with prevalence of increased BPSD burden (preexisting or new symptoms) but with profile of BPSD changes. We confirmed previous findings from the study by Lara *et al*. that showed increased anxiety in MCI patients and apathy in mild-moderate AD patients after COVID-19 quarantine (21). Patients with preserved awareness of the traumatic event and with higher limitations respect to pre-pandemic lifestyle may be at higher risk to develop stress-related symptoms. With disease progression, the heterogeneity of BPSD manifestations reflects the increased multifactorial complexity (40, 41).

Gender effect on the type of BPSD has been demonstrated in AD, with females having more frequently psychotic symptoms and depression (42–44), and males presenting more frequently apathy and aggression (45). Different presentations of BPSD according to gender have already been described and most studies report the prevalence of anxiety and depression among female patients. This gender effect is more evident in mild to moderate stage of the disease and disappear in advanced stage (46). Some other authors report different manifestation of BPSD also in advanced stages of disease with males that exhibited more apathy and sexually inappropriate behavior and females exhibiting more anxiety and sadness (47). Our data are in line with these findings confirming that symptoms of depression and anxiety are more prevalent in women, particularly in the mild stages of the disease (43, 46, 48), while apathy and irritability are more prevalent in males (45).

### Caregivers Distress and Influence on Behavioral and Psychological Symptoms of Dementia

BPSD are the most stressful aspects strongly reducing quality of life for both patients and caregivers. Anxiety and depression accompany caregivers along the entire disease course (49) and caregiver burden is a well-known socially and scientifically recognized aspect (50). Caregiving burden is known to be higher and heavier for women than men (51). In our study we found an increase prevalence of symptoms of anxiety, feeling of helplessness, and anguish reported by caregivers. Increased concern for patient health and increased familial conflicts were also reported. Presence of housemates reduced the risk of depression and conflicts thus indicating that caregiver burden may be mitigated by contrasting loneliness and supporting needs of caring with a network of helps (52).

We found an association between psychological symptoms of anxiety and depression in caregivers and increased BPSD burden. From the results of our survey we could not address the issue of whether increased BPSD are the cause or consequence of caregiver distress, particularly during quarantine when both counterparts have been exposed to similar stressor conditions.

#### Strengths and Limitations

This is the first survey addressing prevalence and type of increase in neuropsychiatric symptoms as acute consequence of imposed isolation due to COVID-19 quarantine in a large population of patients affected by dementia. The sample is large, representative of the most frequent forms of dementia and balanced across groups as far as demographic and clinical variables. Considerations drawn from the results of this study could therefore be extended to community-dwelling subjects affected by dementia. Limitations included the lack of standardized assessment using formal neuropsychiatric rating scales and lack of information on previous BPSD severity and type. This was due to the narrative nature of phone-based interview, the organizational constrains due to the emergency setting, and the need to recruit a large sample in a short time to monitor acutely the neuropsychiatric modifications during quarantine. Moreover, the interview was delivered to caregivers and therefore reports could be influenced by their emotional status and level of distress. However, there are studies confirming the reliability of caregivers reporting BPSD in dementia (53). Another limitation is the absence of information on type of drug prescription modification made in more than one-quarter of patients with BPSD changes. This would have been interesting since use of some drugs classes, such as antipsychotics, modify the risk of stroke and mortality and since an untailored therapeutic plan during quarantine could be partially responsible for BPSD worsening.

## Data Availability Statement

The raw data supporting the conclusions of this article will be made available by the authors, without undue reservation.

## Ethics Statement

The studies involving human participants were reviewed and approved by Ethics Committee of the Coordinating Center (University of Torino on April 7, 2020, n.00150/2020). Written informed consent for participation was not required for this study due to lockdown restrictions and in accordance with the national legislation and the institutional requirements.

## Author Contributions

IR, AB, CM, AC, LB, CC, and VL designed the study and planned centre recruitment. AC, CM, RL, and AB wrote the report. RL and PP did the statistical analyses. ER, VA, VI, NV, FA, IA, PC, MP, IP, RS, DQ, VG, GL, MF, GT, and CF contributed to the interpretation and discussion of results and reviewed the manuscript. All authors contributed to the article and approved the submitted version. 

## Conflict of Interest

The authors declare that the research was conducted in the absence of any commercial or financial relationships that could be construed as a potential conflict of interest.

The handling editor declared a shared affiliation with several of the authors, CM, DQ, and GV, at time of review.

## List of collaborating authors in the SINdem COVID-19 study group

**Aging Brain and Memory Clinic, Department of Neuroscience, University of Torino, Turin, Italy** (Erica Gallo, MD, erica.gallo@unito.it; Alberto Grassini, MD, alberto.grassini@unito.it; Andrea Marcinnò, MD, andrea.marcinno@unito.it; Fausto Roveta, MD, fausto.roveta@unito.it; Paola De Martino, MD paolademartino@unito.it.)

**Regional Neurogenetic Centre ASP Catanzaro, Catanzaro, Italy** (Francesca Frangipane, MD, francesca.frangipane@libero.it; Gianfranco Puccio, MD, puccio@arn.it; Rosanna Colao, MD, ros.colao@gmail.com; Maria Mirabelli, PsyD, mariamirabelli@libero.it)

**Memory Clinic, Fondazione Policlinico Gemelli, Università Cattolica del Sacro Cuore, IRCCS Rome, Italy** (Noemi Martellacci, PsyD, noemi_18@hotmail.com; Federica Lino, PsyD, federica.lino.psy@gmail.com)

**Department of Neuroscience, University of Padua, Padua, Italy** (Stefano Mozzetta, MD, st.mozzetta@gmail.com; Cinzia Bussè, PhD, cinzia.busse@gmail.com)

**CDCD Aulss6 Alta Padovana, Padua, Italy** (Giulia Camporese, MD giulia.camporese@aulss6.veneto.it)

**Department of Biotechnological and Applied Clinical Sciences, Neurological Institute, University of L’Aquila, L’Aquila, Italy** (Simona Sacco, MD, simona.sacco@univaq.it)

**Avezzano Hospital, Avezzano, Italy** (Maria Carmela Lechiara, MD mlechiara@asl1abruzzo.it)

**Department of Neuroscience, Imaging and Clinical Sciences, University G. d’Annunzio, Chieti, Italy** (Claudia Carrarini, MD, claudia.carrarini@live.it, Mirella Russo, MD, mirella.russo92@gmail.com

**Azienda Sanitaria 02 Lanciano Vasto Chieti,** **Italy** (Alfonsina Casalena, MD, alfonsinacasalena@yahoo.it)

**Clinica Neurologica San Salvatore Hospital, L’Aquila, Italy** (Patrizia Sucapane, MD p_sucapane@yahoo.com)

**Division of Neurology, Scientific Institute for Research, Hospitalization, and Care (IRCCS), Foundation “Carlo Besta” Neurological Institute, Milan, Milan, Italy** (Pietro Tiraboschi, MD, Pietro.Tiraboschi@istituto-besta.it, Paola Caroppo, MD, paola.caroppo@istituto-besta.it; Veronica Redaelli, MD, veronica.redaelli@istituto-besta.it, Giuseppe Di Fede, MD giuseppe.difede@istituto-besta.it)

**CDCD Serra Spiga ASP Cosenza, Cosenza, Italy** (Daniela Coppa, MD, dncoppa@gmail.com,

Lenino Peluso, MD dott.pelusolenino@gmail.com)

**CDCD Polistena Laureana ASP Reggio Calabria, Cinquefrondi, Italy** (Pasqualina Insarda, MD linainsarda@tiscali.it)

**CDCD Jonio Sud District ASP Cosenza, Corigliano-Rossano, Italy** (Matteo De Bartolo, MD debartolo.matteo@libero.it)

**First Division of Neurology, University of Campania “Luigi Vanvitelli”, Napoli, Naples, Italy** (Sabrina Esposito, MD sabrina.esposito1@unicampania.it)

**CDCD AORN “Ospedale dei Colli” – CTO, Napoli, Naples**, **Italy** (Alessandro Iavarone, MD alessandro.iavarone@ospedalideicolli.it)

**CDCD DS 50, ASL Napoli 3 Sud, Naples**, **Italy** (Anna Vittoria Marta Orsini, MD annaorsini@virgilio.it

**CDCD Neurologia, University of Campania “Federico II”, Napoli**, **Italy** (Elena Salvatore, MD, e.salvatore@unina.it; Chiara Criscuolo, MD sky569@hotmail.com)

**IRCCS Istituto delle Scienze Neurologiche di Bologna, UOC Clinica Neurologica Rete Neurologica Metropolitana (NEUROMET), Italy** (Luisa Sambati, MD, luisasambati@gmail.com, Rossella Santoro, Ph.D, rossella.santoro@aosp.bo.it)

**AOU Sant’Anna di Icona – Ferrara, Ferrara, Italy** (Daniela Gragnaniello, MD, d.gragnaniello@ospfe.it)

**CDCD Ospedale del Delta, AUSL Ferrara, Ferrara, Italy** (Ilaria Pedriali, MD, ilaria.pedriali@ospfe.it)

**CDCD AUSL Parma, Parma, Italy** (Livia Ludovico, MD, lludovico@ausl.pr.it

**AUO Policlinico Modena, Modena, Italy** (Annalisa Chiari, MD; chiari.annalisa@aou.mo.it)

**UOC Cognitive Disorders and Dementia, Department of Primary Care, AUSL Modena, Italy** (Andrea Fabbo, MD, a.fabbo@ausl.mo.it; Petra Bevilacqua, PsyD, p.bevilacqua@ausl.mo.it; Chiara Galli, PsyD, ch.galli@ausl.mo.it; Silvia Magarelli, Psy.D, s.magarelli@ausl.mo.it)

**A.I.M.A.–sez Parma, Parma, Italy** (Marta Perini Psy.D perini_marta@libero.it)

**Fondazione Santa Lucia IRCCS, Rome, Italy & Menninger Department of Psychiatry and Behavioural Sciences, Baylor College of Medicine, Houston, Tx, USA** (Gianfranco Spalletta, MD, (g.spalletta@hsantalucia.it)

**Fondazione Santa Lucia IRCCS, Rome, Italy** (Nerisa Banaj, MD, n.banaj@hsantalucia.it; Desirée Estela Porcari, PsyD, de.porcari@hsantalucia.it; Giulia Caruso, PsyD g.caruso@hsantalucia.it)

**Dipartimento di Neuroscienze, Università di Roma “Tor Vergata”, Rome, Italy** (Desirée Estela Porcari, PsyD)

**AOU Sant’Andrea, Rome, Italy** (Virginia Cipollini MD, Ph.D, virginiacipollini@uniroma1.it)

**AO San Giovanni Addolorata, Rome, Italy** (Anna Rosa Casini, MD arosa.casini@virgilio.it)

**Campus Biomedico, University of Roma, Rome, Italy** (Francesca Ursini, MD, f.ursini@unicampus.it)

**Department of Neuroscience, University of Roma “La Sapienza”, Rome, Italy** (Giuseppe Bruno, MD, giuseppe.bruno@uniroma1.it)

**Department of Geriatrics Fondazione Poliambulanza di Brescia, Italy** (Renzo Rozzini MD, renzo.rozzini@poliambulanza.it)

**Luigi Sacco Hospital, University of Milano, Milan, Italy** (Michela Brambilla, PsyD, michela.brambilla@libero.it)

**Unit of Neurology, IRCCS San Raffaele Scientific Institute and Vita-Salute San Raffaele University, Milan, Italy** (Giuseppe Magnani, MD, magnani.giuseppe@hsr.it; Francesca Caso, MD; caso.francesca@hsr.it; Edoardo G. Spinelli, MD, spinelli.edoardogioele@hsr.it)

**Unit of Behavioral Neurology IRCCS Mondino Foundation, and Department of Brain and Behavioral Sciences, University of Pavia, Italy** (Matteo Cotta Ramusino MD matteo.cottaramusino@mondino.it; Giulia Perini MD, giulia.perini@mondino.it)

**Department of Experimental and Clinical Medicine, Polytechnic University of Marche, Ancona, Italy** (Simona Luzzi, MD, s.luzzi@staff.univpm.it)

**CDCD Mazzoni Hospital, Ascoli Piceno, Italy** (Gabriella Cacchiò, MD, (cacchiogabriella@tiscali.it)

**CDCD Area Vasta 4, Fermo, Italy** (Rossano Angeloni, MD, rossano.angeloni@sanita.marche.it)

**Geriatric Operative Unit, IRCCS-INRCA, Fermo, Italy** (Cinzia Giuli, PsyD, c.giuli@inrca.it)

**CDCD Area Vasta 3, Macerata, Italy** (Katia Fabi, MD katia.fabi@libero.it)

**Azienda Ospedaliera Marche Nord, Pesaro, Italy** (Marco Guidi, MD, marcoguidi55@gmail.com)

**CDCD San Benedetto del Tronto, Italy** (Cristina Paci, MD, cpaci@libero.it)

**CDCD IRCSS Neuromed di Pozzilli, Isernia, Italy** (Annaelisa Castellano, MD, annaelisacastellano@yahoo.it)

**Neurodegenerative Centre, University of Bari “Aldo Moro”, Bari, Italy** (Elena Carapelle, MD, elecarpi@hotmail.it; Rossella Petrucci, RN, rpetrucci78@gmail.com, Miriam Accogli, ScD)

**CDCD of Casarano, Lecce, Italy (**Gianluigi Calabrese MD, gianluigicalabrese@libero.it **)**

**CDCD DSS of Campi Salentina, Lecce, Italy** (Giovanna Nicoletta Trevisi, MD, trevisigiovanna@libero.it)

**CDCD DSS of Lecce, Lecce, Italy** (Brigida Coluccia MD, colbrig@libero.it)

**CDCD DSS of Maglie, Maglie, Italy** (Antonella Vasquez Giuliano, MD)

**CDCD Ospedale Vito Fazzi Lecce, Italy** (Marcella Caggiula MD)

**Department of Neurology, University of Milano – Bicocca, Milan, Italy** (Fulvio Da Re, MD)

**CDCD PO Santissima Trinità, ASSL Cagliari, Cagliari, Italy** (Antonio Milia, MD, antoniomilia55@gmail.com; Giuseppina Pilia, MD, giusi.pilia77@gmail.com; Maria Giuseppina Mascia, MD, mgmascia@gmail.com)

**CDCD Area Vasta 1, Cagliari, Italy** (Valeria Putzu, MD putzu.valeria@tiscali.it)

**Section of Neurology Department of Biomedicine, Neuroscience and Advanced Diagnostics, University of Palermo, Palermo, Italy** (Tommaso Piccoli, MD tommaso.piccoli@gmail.com, Luca Cuffaro, MD, cuffaro.luca@gmail.com, Roberto Monastero, MD roberto.monastero@gmail.com)

**Neurology and Neurophysiopathology Unit, AOUP “Paolo Giaccone”, Palermo, Italy** (Antonella Battaglia, PsyD, antobatt1994@yahoo.it, Valeria Blandino, PsyD, valeriabl@libero.it, Federica Lupo, PsyD, federicalupo1@gmail.com)

**UO Neurodegenerative Disorders, ASP 2, Caltanissetta, Italy** (Eduardo Cumbo, MD eduardo.cumbo@tiscali.it)

**AOU Policlinico “Vittorio Emanuele”, Catania, Italy** (Luca Antonina, MD antolucaster@gmail.com)

**AO Cannizzaro, Catania, Italy** (Giuseppe Caravaglios, MD giuseppe.caravaglios@gmail.com)

**Psychogeriatric Unit, ASP Messina, Messina, Italy** (Annalisa Vezzosi, MD psicogeriatria@asp.messina.it)

**Neurology I, Department of Neuroscience, Psychology, Drug Research and Child Health, AOU Careggi, Florence, Italy** (Valentina Bessi, MD valentina.bessi@unifi.it)

**CDCD, Neurology I, AOU University of Pisa, Pisa, Italy** (Gloria Tognoni, MD gloria.tognoni@med.unipi.it)

**Geriatric Unit, Department of Clinical and Experimental Medicine, University of Pisa, Pisa, Italy** (Valeria Calsolaro, MD valina82@gmail.com)

**CDCD, Division of Geriatric and Intensive Care Medicine, AOU Careggi and University of Florence, Italy** (Enrico Mossello MD)

**CDCD Territoriale, USL Umbria 1, Perugia, Italy** (Serena Amici, MD, serena.amici@uslumbria1.it, Alberto Trequattrini, MD, alberto.trequattrini@uslumbria1.it; Salvatore Pezzuto, MD, salvatore.pezzuto@uslumbria1.it)

**Department of Medicine, University of Perugia, Perugia, Italy** (Patrizia Mecocci, MD, patrizia.mecocci@unipg.it, Giulia Fichera MD, giuliafcr@gmail.com)

**CDCD AUSSL 7 Pedemontana, Bassano del Grappa, Italy** (Samantha Pradelli Psy.D samantha.pradelli@aulss7.veneto.it)

**CDCD Geriatria, Dolo, Venice, Italy** (Marino Formilan, MD uva.geriatriadolo@aulss3.veneto.it)

**CDCD Geriatric Unit, University of Padua, Padua, Italy** (Alessandra Coin, MD alessandra.coin@unipd.it)

**CDCD AULSS 9 Scaligera, Verona, Italy** (Laura Detogni, MD, laura.detogni@aulss9.veneto.it; Francesca Sala, MD, francesca.sala@aulss9.veneto.it; Giulia Sandri, MD neuropsicologia.villafranca@aulss9.veneto.it)

**CDCD AULSS 2 Marca Trevigiana, Treviso, Italy** (Maurizio Gallucci, MD, maurizio.gallucci@aulss2.veneto.it; Anna Paola Mazzarolo, PhD, annapaola.mazzarolo@aulss2.veneto.it, Cristina Bergamelli, PhD cristina.bergamelli@aulss2.veneto.it)
