# Supplementary material for: Behavioral and Psychological Effects of Coronavirus Disease-19 Quarantine in Patients With Dementia
Source: Front Psychiatry. 2020 Sep 9;11:578015. doi: 10.3389/fpsyt.2020.578015 (PMC7509598; doi:10.3389/fpsyt.2020.578015)
Supplement: Supplementary file 1 [file DataSheet_1.docx]

SURVEY Covid-19 and dementia

Form A

**A.1 Caregiver**

Sex: Male ___ Female ___

Age ________ Education (years)________

Relative of the person with dementia (type):____________________________________________

1. Do you live with the person suffering from dementia?

YES_____

NO_____

1. If so, were you already living together before quarantine?

YES_____

NO_____

1. If not, is the patient institutionalized?

YES_____

NO_____

Have you the possibility to see him regularly

YES____

NO____

1. Are there other people in the house?

YES_____

NO_____

1. Are you working during quarantine?

YES_____

NO_____

1. Has your life changed with quarantine?

NO

YES, I have less time to devote to myself.

YES, I have conflicts with other family members.

7) Has the relationship with your relative been changed?

NO

YES, increase of conflicts

YES, improvement of the relationship

8) Are you concerned about possible consequences of COVID 19 on the patient's health?

YES_____

NO_____

9) Do you think that specific guidance on the prevention of COVID 19 infection in patients with dementia may be useful?

YES_____

NO_____

10) In general, are you experiencing problems in care/therapeutic continuity?

YES_____

NO_____

11) Have you been in the situation to search first aid services (out-of-hours service doctor/ emergency medical care) for yourself or the patient due to the lack of assistance on the territory by the medical practitioner (family doctor) or specialist consultant?

YES_____

NO_____

12) Would you be in favour of the use of tools such as telephones and computers, and use of remote psychological support for outpatient visits in suspended services?

YES_____

NO_____

13) Did you receive any help or support during the COVID emergency?

NO

Yes, by the family

Yes, from the neighbours/friends

Yes, from the Municipality

Yes, from volunteering (associations, parishes, red cross, others)

14) Did you feel isolated or abandoned during the COVID emergency?

YES_____

NO_____

15 Did you feel overwhelmed or helpless?

YES_____

NO_____

16 Did you present

anguish Yes ___No___

depression Yes ___No___

anxiety Yes ___No___

irritability Yes ___No___

**A.2 Patient**

Age__________________

Sex: Male ___ Female ___

Type of dementia: ____________________________________________________________

Year of dementia onset____________

Quarantine start date: ___ / ___ / ___

1. Before quarantine the patient was used to get out of the house

Yes, independently ___

Yes, accompanied, ___

No ___

2) What is the stage of the disease?

Mild

Moderate

Severe

Bedridden

3) Have you noticed patient’s behavioural changes since the beginning of the quarantine?

YES_____

NO_____

If Yes, what are the behavioral symptoms that have increased in intensity but were already present before quarantine?

o Apathy (reduction of initiative)

o Anxiety

o Depression

o Sleep disorders

o Delusions (i.e. the patient has thoughts that someone wants to hurt him, steal from him, kill him, etc.).

o Hallucinations (the patient sees or hears things or people that are not there)

o Irritability

o Aggressiveness

o Wandering (the patient walks around aimlessly and irrepressibly)

o Agitation

o Change in appetite

4) Did you notice any new behavioral symptoms, which were not present before the quarantine period?

o There were no new symptoms detected

o Apathy (reduction of initiative)

o Anxiety

o Depression

o Sleep disorders

o Delusions (the patient has thoughts that someone wants to hurt him, wants to rob him, kill him etc.)

o Hallucinations (seeing and hearing things or people who are not there)

o Irritability

o Aggressiveness

o Wandering (walking around aimlessly and irrepressibly)

o Agitation

o Change in appetite

5) Because of these disorders, was it necessary to change the medication therapy?

YES_____

NO_____

6) Have you noticed any changes in the patient’s physical performances in the last month?

o I think he/she walks better than before

o No

o He/she walks slower

o He/she can't stand up independently from the chair anymore.

o He/she doesn't get up from bed anymore

7) Have you noticed any changes in the patient’s cognitive symptoms that were present before quarantine?

NO_______

YES______

o Increased confusion

o Greater forgetfulness

o More difficulty in finding the correct words

o In-door space disorientation and difficulties of house recognition

o Disorientation in time

o Loss of recognition of family members

o Troubles of self-recognition in the mirror

8) Do you think the disease has progressed faster during the quarantine period?

YES_____

NO, I think it's taking its natural course______

9) Is the patient aware of the COVID emergency?

YES_____

NO_____

Date .......................................................................................
