# Supplementary material for: Behavioral and Psychological Effects of Coronavirus Disease-19 Quarantine in Patients With Dementia
Source: Front Psychiatry. 2020 Sep 9;11:578015. doi: 10.3389/fpsyt.2020.578015 (PMC7509598; doi:10.3389/fpsyt.2020.578015)
Supplement: Supplementary file 2 [file Table_1.docx]

Supplementary table: Frequency distribution and interval confidence of worsened preexisting and new BPSD in the entire patients’ sample and divided by disease type.

| **Patients** | **Total (n=4913)** | **AD (n=3372)** | **DLB (n=360)** | **FTD (n=415)** | **VD (n=766)** |
| --- | --- | --- | --- | --- | --- |
| **Worsening, % (IC 95%)** | | | | | |
| Apathy | **17.9**  (16.9-19.0) | **17.8**  (16.6-19.2) | **21.9**  (17.8-26.6) | **19.8**  (16.1-24.0) | **15.5**  (13.1-18.3) |
| Anxiety | **15.1**  (14.2-16.2) | **15.7**  (14.5-17.0) | **18.6**  (14.8-23.0) | **12.5**  (9.6-16.2) | **12.5**  (10.3-15.1) |
| Depression | **13.0**  (12.1-14.0) | **12.4**  (11.3-13.6) | **18.0**  (14.3-22.4) | **12.0**  (9.2-15.7) | **14.0**  (11.6-16.7) |
| Sleep disorder | **12.5**  (11.6-13.5) | **11.5**  (10.5-12.6) | **21.9**  (17.8-26.6) | **13.5**  (10.4-17.2) | **12.0**  (9.8-14.6) |
| Delusions | **5.1**  (4.5-5.8) | **4.4**  (3.8-5.2) | **10.0**  (7.2-13.7) | **6.3**  (4.2-9.2) | **5.2**  (3.8-7.1) |
| Hallucinations | **5.1**  (4.5-5.8) | **4.1**  (3.5-4.9) | **18.6**  (14.8-23.0) | **3.4**  (1.9-5.7) | **4.2**  (2.9-5.9) |
| Irritability | **20.9**  (19.8-22.1) | **20.6**  (19.2-22.0) | **20.2**  (16.3-24.8) | **21.7**  (17.9-26.0) | **22.1**  (19.2-25.2) |
| Aggressiveness | **9.6**  (8.8-10.4) | **8.9**  (8.0-10.0) | **10.2**  (7.4-14.0) | **11.1**  (8.3-14.6) | **11.2**  (9.1-13.7) |
| Wandering | **6.4**  (5.8-7.1) | **6.1**  (5.3-6.9) | **3.9**  (2.2-6.6) | **10.1**  (7.5-13.5) | **7.2**  (5.5-9.3) |
| Agitation | **16.0**  (15.0-17.0) | **15.0**  (13.8-16.2) | **20.5**  (16.5-25.1) | **19.5**  (15.9-23.7) | **16.2**  (13.7-19.0) |
| Change of appetite | **5.7**  (5.1-6.4) | **5.3**  (4.6-6.1) | **6.1**  (3.9-9.2) | **8.7**  (6.2-11.9) | **6.0**  (4.5-8.0) |
| **New onset, % (IC 95%)** | | | | | |
| Apathy | **4.4**  (3.9-5.1) | **4.6**  (3.9-5.3) | **4.4**  (2.6-7.2) | **3.9**  (2.3-6.3) | **4.2**  (2.9-5.9) |
| Anxiety | **3.4**  (2.9-3.9) | **3.5**  (2.9-4.2) | **3.3**  (1.8-5.9) | **2.4**  (1.2-4.5) | **3.4**  (2.3-5.0) |
| Depression | **3.2**  (2.8-3.8) | **3.5**  (2.9-4.2) | **1.7**  (0.7-3.8) | **1.7**  (0.7-3.6) | **3.5**  (2.4-5.2) |
| Sleep disorder | **5.5**  (4.9-6.2) | **5.7**  (4.9-6.5) | **3.0**  (1.6-5.5) | **4.6**  (2.9-7.2) | **6.5**  (4.9-8.6) |
| Delusions | **2.6**  (2.2-3.1) | **2.4**  (1.9-3.0) | **3.3**  (1.8-5.9) | **2.4**  (1.2-4.5) | **3.1**  (2.1-4.7) |
| Hallucinations | **2.5**  (2.1-3.0) | **2.6**  (2.1-3.2) | **2.8**  (1.4-5.2) | **1.4**  (0.6-3.3) | **2.6**  (1.6-4.1) |
| Irritability | **5.4**  (4.7-6.0) | **5.8**  (5.0-6.6) | **4.2**  (2.4-6.9) | **3.6**  (2.1-6.0) | **5.1**  (3.7-7.0) |
| Aggressiveness | **3.4**  (2.9-3.9) | **3.4**  (2.8-4.0) | **3.0**  (1.6-5.5) | **3.4**  (1.9-5.7) | **3.7**  (2.5-5.3) |
| Wandering | **2.3**  (1.9-2.8) | **2.4**  (2.0-3.0) | **1.1**  (0.4-3.0) | **1.9**  (0.9-3.9) | **2.7**  (1.7-4.2) |
| Agitation | **4.7**  (4.2-5.4) | **5.0**  (4.3-5.8) | **3.9**  (2.2-6.6) | **3.6**  (2.1-6.0) | **4.6**  (3.2-6.4) |
| Change of appetite | **4.1**  (3.6-4.7) | **4.4**  (3.7-5.2) | **2.8**  (1.4-5.2) | **3.9**  (2.3-6.3) | **3.8**  (2.6-5.5) |
